# Preliminary validation of the use of IgG antibody response to *Anopheles* gSG6-p1 salivary peptide to assess human exposure to malaria vector bites in two endemic areas of Cameroon in Central Africa

**DOI:** 10.1371/journal.pone.0242510

**Published:** 2020-12-31

**Authors:** Glwadys Cheteug, Emmanuel Elanga-Ndille, Christiane Donkeu, Wolfgang Ekoko, Martine Oloume, Estelle Essangui, Philippe Nwane, Sandrine Eveline NSango, Josiane Etang, Samuel Wanji, Lawrence Ayong, Carole Else Eboumbou Moukoko

**Affiliations:** 1 Malaria Research Unit, Centre Pasteur Cameroon, Yaounde, Cameroon; 2 Department of Microbiology and Parasitology, Faculty of Sciences, University of Buea, Buea, Cameroon; 3 Centre for Research in Infectious Diseases, Yaounde, Cameroon; 4 Department of Animal Biology and Physiology, Faculty of Sciences, University of Yaounde, Yaounde, Cameroon; 5 Parasitology and Entomology Research Unit, Department of Animal Biology and Organisms, Faculty of Sciences, University of Douala, Douala, Cameroon; 6 Department of hematology, Centre Pasteur of Cameroon, Yaoundé, Cameroon; 7 Biological Sciences Department, Faculty of Medicine and Pharmaceutical Sciences, University of Douala, Douala, Cameroon; 8 Laboratory of Parasitology, Mycology and Virology, Postgraduate Training Unit for Health Sciences, Postgraduate School for Pure and Applied Sciences, University of Douala, Douala, Cameroon; 9 Organisation de Coordination pour la Lutte contre les Endemies en Afrique Central, Yaounde, Cameroon; Arizona State University, UNITED STATES

## Abstract

The specific immune response to the *Anopheles* salivary peptide could be a pertinent and complementary tool to assess the risk of malaria transmission and the effectiveness of vector control strategies. This study aimed to obtain first reliable data on the current state of the *Anopheles* gSG6-P1 biomarker for assess the level of exposure to *Anopheles* bites in high malaria endemic areas in Cameroon. Blood smears were collected from people living in the neighborhoods of Youpwe (suburban area, continental) and Manoka (rural area, Island), both areas in the coastal region of Cameroon. Malaria infection was determined using thick blood smear microscopy, whereas the level of specific IgG response to gSG-P1 peptide was assessed by enzyme-linked immunosorbent assay from the dried blood spots. Of 266 (153 from Youpwe, 113 from Manoka) malaria endemic residents (mean age: 22.8±19.8 years, age range: 6 months–94 years, male/female sex ratio: 1/1.2, with Manoka mean age: 23.71±20.53, male/female sex ratio:1/1.13 and Youpwe mean age: 22.12±19.22, male/female sex ratio 1/0.67) randomly included in the study, *Plasmodium* infection prevalence was significantly higher in Manoka than in Youpwe (64.6% vs 12,4%, p = 0.0001). The anti-gSG6-P1 IgG response showed a high inter-individual heterogeneity and was significantly higher among individuals from Manoka than those from Youpwe (p = 0.023). Malaria infected individuals presented a higher anti-gSG6-P1 IgG antibody response than non-infected (p = 0.0004). No significant difference in the level of specific IgG response to gSG-P1 was observed according to long lasting insecticidal nets use. Taken together, the data revealed that human IgG antibody response to *Anopheles* gSG-P1 salivary peptide could be also used to assess human exposure to malaria vectors in Central African region. This finding strengthens the relevance of this candidate biomarker to be used for measuring human exposure to malaria vectors worldwide.

## Introduction

Although the global malaria incidence and mortality due to *Plasmodium falciparum* decreased by 18% with the largest reductions recorded in Southeast Asia, Latin America, and Africa following the introduction of artemisinin-based combination therapies (ACTs) [1, 2], this disease still remains a public health problem, with 228 million of clinical cases and 405,000 deaths recorded worldwide in 2018 [3]. The vast majority of these cases are reported in sub-Saharan African countries, especially among young children and pregnant women. In the same period, 3% of the worldwide cases were reported in Cameroon, where malaria remains highly endemic [3].

Assessment of malaria transmission intensity (MTI) by mosquitoes is an important determinant of the malaria’s burden estimation and evaluation of effectiveness of control and prevention strategies. It is central to efforts to control and eradicate malaria, and currently estimated by the entomological inoculation rate (EIR) usually interpreted as the number of infective bites received by an individual per unit of time [4]. Unfortunately, this entomological parameter has significant drawbacks and limitations such as inaccuracy due to micro-heterogeneity of malaria transmission, especially in areas of low transmission [5, 6]. Furthermore, the EIR may lack sensitivity due to the limited number of *Plasmodium*-positive samples to accurately estimate the sporozoïte index [7, 8]. The human-landing catches (HLC) regarding as the “gold standard” method [9] remain the best method and, has been used by in man studies to estimate the human biting rate (HBR) which is a key determinant of the EIR [10, 11]. However HLC is labour-intensive, is subjected to collector bias and, has come under scrutiny because of ethical concerns as it exposes collectors to potentially infectious mosquito bites (Increasingly, ethical review committees are reluctant to approve studies that include the use of HLCs) [12, 13]. While other alternative method that do not require human exposure, as Centers for Disease Control and Prevention light trap catches (LTC) for estimating biting rates exist [14, 15], these also present the disadvantages: LTC trap generally give low mosquito density, difficult acceptability and community involvement, theft of the device by the population [11, 14, 16, 17].

In this context, there was an increased need to develop novel approaches for an efficient assessment of malaria transmission intensity. Previous studies have shown that immunological markers based on the quantification of human antibody responses to malaria vector salivary antigens could be useful for measuring MTI [18–22]. More specifically, IgG antibody response to the gSG6-P1 salivary peptide (*An*. *gambiae* Salivary Gland Protein-6 peptide 1) has been validated as a relevant biomarker of *Anopheles* bites in various settings including West and East Africa [23–29], America [30] and Asia [31, 32]. Moreover, in Africa, antibody response to gSG6-P1 salivary peptide was also found relevant for detecting human exposure to malaria vectors in the context of low and seasonal mosquito densities [24, 26, 27–29]. Interestingly, immune specific response to this biomarker was reported to be a pertinent tool to assess the efficacy of malaria vector control strategies in several countries in West and East Africa [24, 33, 34].

To our knowledge, there are currently no published studies that have scientifically evaluated the useful of this biomarker to measure malaria transmission intensity in Central African region in the published literature. Therefore, to provide concrete data on the use of human IgG Ab response to the gSG6-P1 salivary peptide as an epidemiological tool for assessing exposure of human population to malaria vectors in Cameroun, we conducted a prospective population based study to estimate the level of exposure to *Anopheles* bites in a continental suburban area and rural island in the coastal region of Cameroon. It will be suitable to compare the exposition to Anopheles bites and malaria transmission between such distinct areas, because it’s important to know, if all strategies used in the fight against malaria are working as well as in rural area than in sub-urban area. Because, the distinct geographical nature of the two sites (isolated Island and continental area) will induce difference in the malaria transmission risk.

## Materials and methods

### i) Study design and population

A cross-sectional study was conducted during the small rainy season in May 2017 and was carried out in 2 localities (Manoka Island a rural area and Youpwe a sub-urban continental area) in the coastal region of Cameroon.

The Island of Manoka (03 °47'N; 9°39'E) is located in the archipelago of Manoka in the rural area of the 6th district of Douala, the most industrial city of Cameroon. This location is populated by around 40, 000 inhabitants. The main activities on this island are: fishing, production and trade of smoking fish products. Habitations are in temporary materials (houses built on stilts) with opened spaces between the walls of the roof. The hydrographic network is made up of many temporary streams: sources, pools and swamps that also constitute potential breeding sites of mosquitoes. The environment is predominantly marshy here.

Youpwe is located in the sub-urban area in the continental zone, the 6th district of Douala (4°00'N; 9°42'E), the economic capital of Cameroon. An estimated 3,200 people populated this neighborhood. Youpwe serves as a crossroad for trade activities between Douala and the Manoka Island. The main activity here is the general trade, and the selling of products coming from Manoka: fresh and smoked fish, crabs, fresh and dried shrimps. Habitations are mostly built with cement and overall there is no space between the roof and the walls. The environment is made up with many water collections observed: ponds, streams, reserves for household activities and they constitute the mainly breeding site for mosquitoes.

The populations of each of the study sites were sensitized a week earlier by community relay agents who invited them to come to the hospital (health district) on a date set in advance. Once at the hospital, after the study had been presented to the populations, all individual who met the eligibility conditions and agreed to participate in the study were recruited after signing the informed consent. To limit the selection and information biases, participants were enrolled consecutively and participation in the study was voluntary.

The target population were all local residents who gave consent to attend health district of the locality to attend health district of the locality. Eligibility for inclusion as defined as local resident who had not travelled out of the study site within the last 3 weeks. Pregnant women, children under 6 months and individual with severe clinical signs of malaria as defined by WHO, were excluded.

This study was conducted in accordance with ethics directives related to research on humans in Cameroon. The study received an ethical clearance from the National Committee of Ethics for Research for Human Health (N ° 2015 / 08/622 / EC / CNERSH / SP) and was approved by the Cameroonian Ministry of Public Health and, administrative authorization was obtained from all the localities. Before enrollment and the administration of questionnaire, subjects were informed on the purpose and process of the investigation (background, goals, methodology, study constraints, data confidentiality, and rights to opt out from the study), and a signed informed consent was obtained from all those who agreed to participate in the study in accordance with the Helsinki Declaration. Participation was voluntary, anonymous and without compensation. All patients were free treated in accordance to the treatment guidelines from the Cameroon National Malaria Control Program.

### ii) Study questionnaire

They were open-ended (OEIQ) and closed-ended (CEIQ) interview questions, including a single answer, and multiple choices questions. Data collection sheets were used to collect data on socio-demographic characteristics (age, gender, place and year of birth, education and occupation and family and socio-economic background). The second part focused on i) possession and use of LLINs, ii) use of insecticide products (such as sprays or spirals).

### iii) Blood sampling and parasite density

Venous blood samples (3 ml) were collected into EDTA vacutainer tubes and 50 microL were spotted on Whatman filter papers (3M) and stored at 4° until use for the quantification of the level of human IgG Ab response to the *Anopheles* gSG6-P1 salivary peptide. The presence of *P*. *falciparum* and other species of malaria parasites was detected by RDT testing (SD Bioline Malaria Ag *P*.*f*./Pan) and thin blood smears. *P*. *falciparum* parasitaemia was determined by microscopic examination of Giemsa-stained thick blood smears. Parasite density was determined on the basis of the number of parasites per 200 leukocytes on a thick film, assuming total leukocyte counts of 8,000 cells/microL of whole blood.

### iv) Determination of anti-*Anopheles* gSG6-P1 salivary peptide IgG Ab responses

For each blood sample, blood spotted on Whatman (3M) filter papers was collected and analyzed by direct Enzyme-Linked Immunosorbent Assay (ELISA) to estimate total antibody levels against *Anopheles* gSG6-P1 salivary peptide.

For *Anopheles*-specific salivary peptide gSG6-P1, the recombinant antigen sequence identification was designed using bioinformatics as previously described by Poinsignon in 2008 [24]. It was synthesized and purified (> 95%) by Genepep SA (Montpellier, France). Before use, the lyophilized peptide was suspended in ultrapure water and stored in aliquots at -20 °C until use.

The level of IgG Ab response to gSG6-P1 salivary peptide was measured on whole blood elute from a dry spot as previously described by Dramé *et al* [34]. Briefly, 96-wells microplates (F96 Maxisorp, Thermo scientific) were pre-coated with the gSG6-P1 salivary peptide (20 μg/well of antigen) in PBS (1 M, pH 7.4), using 100 μL/well for 2 h 30 min at 37°C., Plate wells were then blocked for 45min at 37°C with 300 μL/well of Pierce Protein-Free PBS blocking buffer, pH 7,4. Individual eluated blood diluted at 1/50 (in PBS-Tween 1%). Individual eluated blood diluted at 1/50 (in PBS-Tween 1%) was then deposited in triplicate in the microplates (2 well with antigen and called “Ag+” and one well without antigen and called “Ag-”) and incubated at 4°C overnight with 100 μL/well. Plates were then incubated with 100 μL/well of Biotin Mouse Anti-Human IgG the secondary Ab solution (BD Pharmingen^™^) at 1/2 000 dilution in PBS/Tween 1% for 1h30min at 37°C. Streptavidine-Peroxydase conjugate (GE Healthcare UK) was then added at 1/2000 dilution and incubated for 1h at 37°C with 100 μL/well. Finally, the plates were subsequently incubated with 100 μL/well of 2,2-azino-bis (3-ethylbenzthiazoline 6-sulfonic acid) diammonium (ABTS) (Thermo scientific) substrate solution (0.05 M citrate buffer, pH = 4) supplemented with 10 μL of 30% of oxygenate water-H_2_O_2_) for 2 hours in the dark and at room temperature for the colorimetric development, then optical density (OD) was read at 405 nm.

Individual results were expressed as the ΔOD value: ΔOD = ODx—ODn, where ODx represents the mean of individual OD in both antigen wells and ODn the individual OD in the well without gSG6-P1 antigen (to remove in each sample all non-specific reaction and background). For each sample, the experiment was validated only if the coefficient of variation between the two Ag+ well (%CV) was ˂20%. Samples with CV ˃20% were re-analysed.

Blood sample (34) from European volunteer blood donors (From “*Etablissement Française du Sang—Normandie”*, *coming from France; 609 chemin de la Bretèque—BP*. *558–76235 BOIS GUILLAUME Cedex*) with no travel history to malaria endemic countries were used as negative controls. Each ELISA experiment was validated by including on each plate three blank wells containing only PBS-Tween 1%in place of blood plasma elute. The cut-off value was defined as a mean OD of negative control plus three times standard deviation (SD).

### v) Statistical analysis

Categorical variables were expressed as frequencies, whereas numerical variables were presented as means+/- Standard deviation (SD) or 95% CI (95% confidence interval) if they were normally distributed. The exact Fisher test was used to compare qualitative variables. After checking the non-Gaussian distribution, the non-parametric Mann-Whitney U-test was used to compare antibody levels between two independent groups. Only p *<*0.05 values were considered significant. All statistical analyses were performed using GraphPad Prism5 software (San Diego, CA, USA) and Stata software (version 11 SE).

## Results

### Characteristics of participants in population survey

A total of 266 individuals from whom blood samples collected were included in the two areas, 153 (57.5%) selected in suburban area (Youpwe) and 113 (42.5%) in rural area (Manoka-Island) (Table 1).

**Table 1:** Basic characteristics of population survey.

	Youpwe	Manoka	Df*[95%CI]	P-value
	n = 153	n = 113		
**Mean stay duration in the study area (SD), years**	8.2 (±8.6)	12.4 (±15.7)	/	0.210
Mean Age (SD)*, years	22.1 (±19.2)	23.7 (±20.5)	/	0.565
**Sex, n(%)**				
Female	92 (60.1)	53 (46.9)	1.70 [1.05–2.79]	0.032
Male	61 (39.9)	60 (53.1)		
**School-Education, n(%)**				
Out-of-school	17 (11.1)	18 (15.9)	4.82 [-3.40–13.04]	0.2525
Primary	42 (27.5)	46 (40.7)	13.26 [1.82–24.70]	0.0231
Secondary	77 (50.3)	42 (37.2)	13.16 [1.07–25.25]	0.0329
University	17 (11.1)	7 (6.2)	4.9 [-2.05–11.88]	0.1665

Data are number and/or proportion (%), unless otherwise indicated;

*, SD: Standard deviation, Df: Difference between fraction (only for Sex and school-education), 95%CI: 95% confidence interval of difference, P-value show the statistical significant or no between Youpwe and Manoka.

The mean length of residence of the individuals surveyed in the study area was higher in Manoka Island compared to Youpwe and the mean of this variable was not significantly different (p = 0.210). Overall, no significant difference was observed for mean age between Youpwe’s population and Manoka’s population (p = 0.565). The entire age range was 6 months to 94 years for Youpwe’s population and 6 months to 85 years Manoka’s population. In each locality, the mean age was 22.12±19.2 with 95%CI (19.05–25.19) and 23.71±20.5 with 95%CI (19.88–27.53) in Youpwe and Manoka Island respectively. Youpwe was predominantly female (92, 60.1%) while, in Manoka Island men accounted for more than half of the population (60, 53.1%) and this difference was statistically significant (p = 0.032). Overall, no difference was observed for an educational level between the two localities (p = 0.05) in our study population, however this difference was significant when comparing individuals one to another in each locality for primary and secondary school level. (Table 1).

### Preventive measures against malaria vectors

Both populations used numerous malaria prevention strategies, including long lasting insecticidal nets (LLINs), ventilation, coils and spray bombs (Table 2). It’s important to mention that, the study were carry out in 2017 and the last mass campaign of LLINs by National Malaria Control Program (NMCP) in the two localities was in 2015.

**Table 2:** Malaria preventive measure according to each area.

	Youpwe n = 153	Manoka n = 113	Df*[95%CI]	P-value
LLINs*, **n (%)**				
LLINs possession	132 (86.3)	93 (82.3)	3.97 [- 4.81–12.75]	0.3750
LLINs Use	117 (88.6)	89 (95.7)	/	0.0868
*All the time*	86 (73.5)	53 (59.6)	13.95 [1.04–26.87]	0.0342
**Insecticide product Use**^$^, **n (%)**	77 (50.3)	50 (44.3)	6.08 [- 6.07–18.23]	0.330
**Ventilation, n (%)**	114 (74.5)	1 (0.9)	/	< 0.0001
**Types of houses, n (%)**				
Cement	137 (89.5)	3 (2.7)	/	<0.0001
Houses built on stilts	16 (9.8)	110 (97.3)	/	<0.0001
**Vectors exposure, n (%)**				
Swampy soils	98 (64.1)	113 (100)	/	<0.0001
Spaces between the roof and walls	64 (41.8)	110 (97.4)	/	<0.0001

Data are number and proportion (%);

*, LLINs, Long lasting insecticidal nets;

^$^,Spray bombs, and coils, Df: Difference between fraction, 95%CI: 95% confidence interval of difference, /, overview (%95CI not available), P-value show the statistical significant or no between Youpwe and Manoka.

Overall, 84.6% of responders reported to have LLINs with a higher proportion of individuals reporting LLINs ownership in Youpwe population (86.3% of *vs* 82.3%, p = 0.3750) and, among them 95.7% declared use of LLINs more in Manoka than in Youpwe but this difference was not statistically significant (p = 0.0868). Additionally, those who use treated nets have been reported to use them every night (all the time) in most of responders, more by Youpwe’s population than Manoka’s population (p = 0.0342). No significant difference was observed when comparing reports on the frequency of LLINs use for children compared to adults. Men reported more LLINs use than women (56.2% vs 43.8%, p = 0.252) in the Manoka Island, whereas in Youpwe women reported more sleep under ITNs than men (63.2% vs 36.8%, p = 0.1761) but this difference was not statistically significant. The environment was mainly swampy and the houses with spaces between the walls and the roof were more important in Manoka compared to Youpwe and, this difference was statistically significant (p<0.0001).

### Clinical and parasitological characteristics

As shown in Table 3, 38.0% of participants with an axillary temperature ≥ 37.5°C (fever), with 37.9% in Youpwe and 38.1% in Manoka Island. No fever cases (temperature less than 37.5°C) accounted for the rest of the participants.

**Table 3:** Clinical and parasitological data of the respondents.

	Youpwe	Manoka	Total	P-value
	n = 153	n = 113	n = 266	
**Clinical status, n (%)**				
Fever	58 (37.9)	43 (38.1)	101 (38.0)	Reference
No fever	95 (62.1)	70 (61.9)	165 (62.0)	1.000
**Malaria diagnostic, n (%)**				
RDT	18 (11.76)	71 (62.8)	89 (33.45)	˂0.0001
Microscopy	7 (4.5)	24 (21.2)	31 (11.6)	˂0.0001
Over all infected (RDT and/or microscopic)	19 (12.4)	73 (63.7)	92 (34.6)	˂0.0001
< 5 years	1 (5.3)	9 (12.3)	10 (10.9)	Reference
[5–16 years]	10 (52.6)	32 (43.8)	44 (47.8)	0.668
[16–60 years]	7 (36.8)	25 (34.2)	33 (35.9)	0.654
≥ 60 years	1 (5.3)	7 (9.6)	8 (8.7)	0.994

Data are number and proportion (%), P-value show the statistical significant or no between Youpwe and Manoka.

Out of the 266 individuals recruited for the study, 89 (33.45%) were detected by RDT positively infected with *Plasmodium*, and the prevalence of infection according to each locality was 11.76% in Youpwe and 62.8% in the Manoka Island. Diagnosis by microscopy generated an overall prevalence of 11.6% with 4.57% in Youpwe and 21.2% in Manoka Island. Taking both RDTs and microscopy together, the overall prevalence was 34.6% (92/266) and the majority of infected people were found in the Island of Manoka, with a prevalence of 63.7% vs 12.4% in Youpwe. (OR = 12.7, 95% CI: 6.80–23.62), p <0.0001) (Table 3).

### Immunological responses to *Anopheles* gSG6-P1 antigen and human exposure to *Anopheles* bites

**Fig 1:**
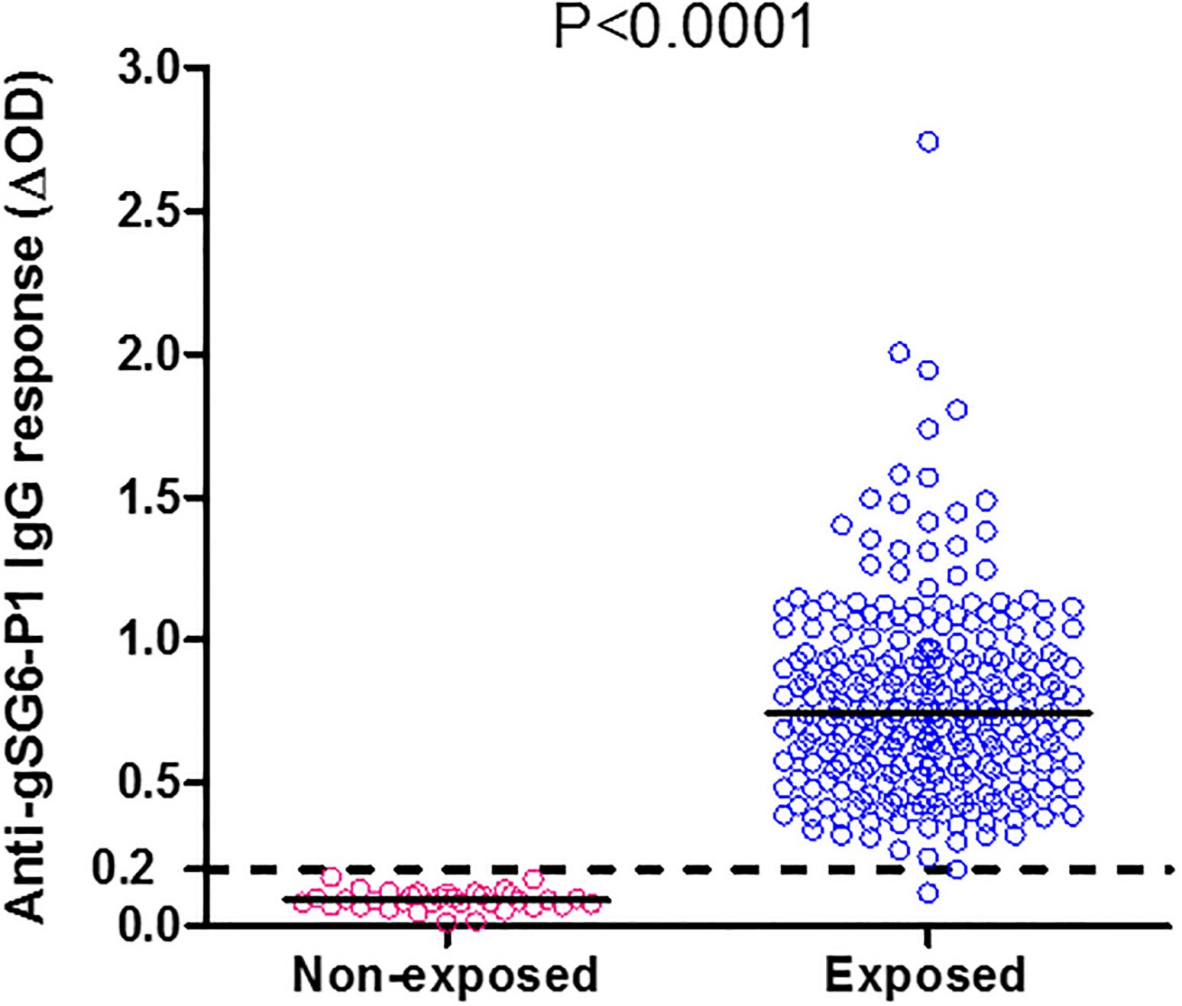
Level of anti-gSG6-P1 IgG response according to exposed and not-exposed individuals. Dot plots show the individual specific IgG level (ΔOD value) to gSG6-P1 between the non-exposed and the exposed individuals, and bars indicate the median value for each group. The Cut-off is represented by dotted line and 99% of the study population were up to the cut-off value. The difference was statistically significantly high in exposure individual (P < 0.0001, non-parametric Mann-Whitney U-test).

To determine if IgG antibody response to gSG6-P1 salivary peptide constitutes tool for detecting human exposure to *Anopheles* bites in Cameroon, analyses were performed based on specific blood antibody levels and positivity for individuals exposed to *Anopheles* bites and European negative controls. As shown in Fig 1, Antigens were differential recognized by the different samples, with the median value of the specific IgG response from exposed individuals (ELISA median ΔOD: 0.740, 95%CI: 0.7438–0.8245) above 4 fold higher than the cutoff value (dotted line on the Fig 1) calculated from the European negative controls (ELISA median ΔOD: 0.093, 95%CI: 0.0811–0.1046) and, the difference between exposure individuals of the study site and European negative controls was statistically significant (p<0.0001).

### Immunological responses to *Anopheles* gSG6-P1 antigen and endemicity area level

**Fig 2:**
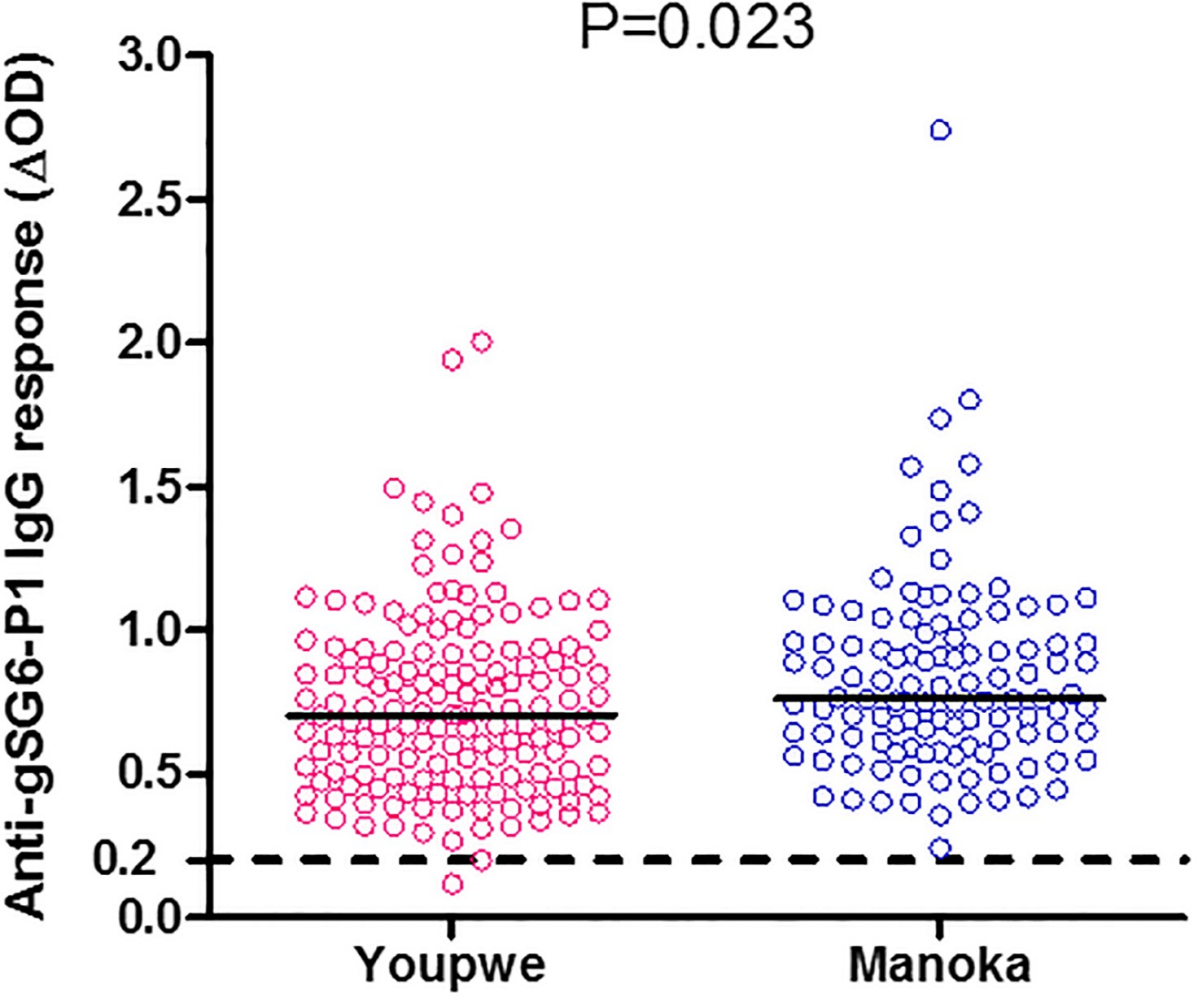
IgG specific Ab response to gSG6-P1 peptide according to localities. Individual ΔOD results are shown, bars indicate median value for each locality. The horizontal black dotted line represents the cut-off of IgG response. Statistical significance between the localities is indicated (non-parametric Mann-Whitney U-test).

To determine if IgG Ab response to gSG6-P1 peptide is associated to the level of exposure to Anopheles bites, we compared the level of this response in Youpwe and Manoka given that since in a preliminary study reported higher exposure to Anopheles bites (aggressiveness) in Manoka (HBR = 20.29 b/p/n) than in Youpwe (HBR = 14.50b/p/n) [35]. The level of antibody responses to the gSG6-P1 salivary peptide was compared between the two study sites in all individuals. Our results as it can be observed in Fig 2, indicates that the level of specific IgG Ab in Manoka Island (median ΔOD: 0.762, 95%CI: 0.772–0.901) is significantly higher than in Youpwe (ELISA median ΔOD: 0.707, 95%CI: 0.694–0.796). Moreover, our results saw a high inter-individual heterogeneity of the IgG response to gSG6-P1 antigen among population of each locality. In the other hand, no significant associations were observed between the level of IgG Ab response to gSG6-P1 peptide according to gender or age.

### Immunological responses to *Anopheles* gSG6-P1 antigen and malaria infection

**Fig 3:**
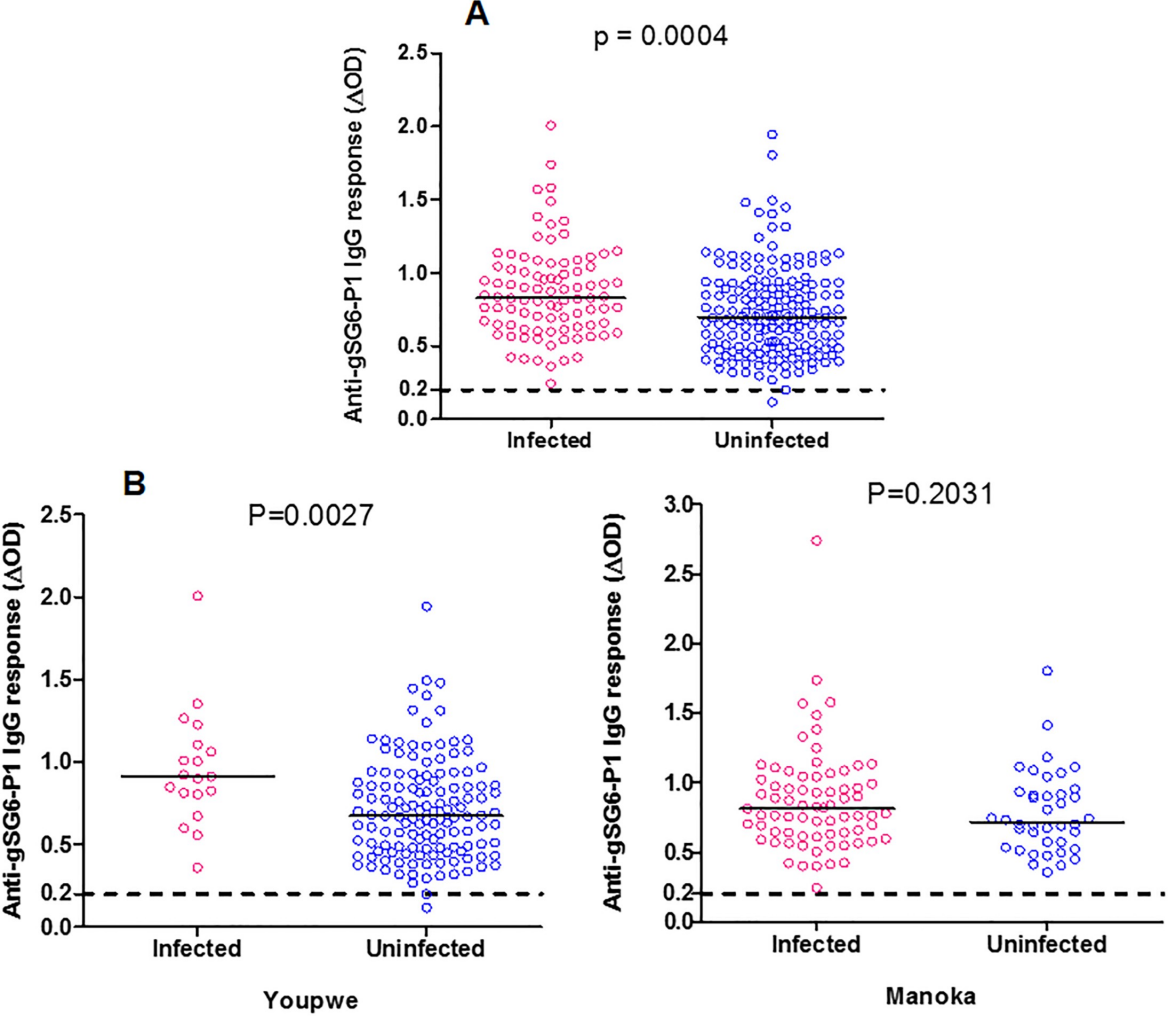
IgG specific Ab response to gSG6-P1 peptide IgG according to malaria infection in whole population (A) and in each locality (B, C). Specific IgG responses are shown (ΔOD) for *Plasmodium* infected and uninfected individual, bars indicate median value for each group. The Cut-of is represented by dotted line. Statistical significance between the infected and uninfected group is indicated (non-parametric Mann-Whitney U-test) in whole population (A), and in each locality (B, C).

To investigate if the level of specific IgG Ab response to gSG6-P1 peptide also depended on infection we compared the specific blood antibody levels and positivity for *Plasmodium* infected population (RDT and/or microscopy positives) and, non-infected population (negative to both tests) (Fig 3A). There was a significant difference of specific IgG level between infected versus not infected individuals in Youpwe whereas this difference not exist in Manoka (Fig 3B & 3C).

As shown in Fig 3A, the highest IgG Ab response to gSG6-P1 peptide observed in infected individuals (median ΔOD: 0.83, 95%CI: 0.800–0.930) compared to non-infected (median ΔOD: 0.69, range: 95%CI: 0.685–0.775) in overall studied population and, this was significantly different (p = 0.0004). Positivity for infection (median ΔOD: 0.91, 95%CI: 0.78–1.13) was also significantly associated with high levels of IgG response compared to non-infected (median ΔOD: 0.67, 95%CI: 0.66–0.76) in Youpwe (p = 0.0027) but not in Manoka (p = 0.203) (Fig 3B & 3C).

### Immunological responses to *Anopheles* gSG6-P1 antigen and preventive strategies against *Anopheles* bites

To investigate if the level of specific IgG Ab response to gSG6-P1 salivary peptide constitutes tool for evaluate the impact of preventive measures against malaria infection, analyses revealed in overall population no significantly difference observed according to the use of anti-mosquitoes bites strategies. No difference was observed between individuals that reported using LLINs and those that did not in each area. The level of anti-gSG6-P1 peptide IgG Ab response was higher among people who reported using LLINs frequently (ELISA median ΔOD: 0.76, 95%CI: 0.751–0.848) than those who did not use LLINs (ELISA median ΔOD: 0.708, 95%CI: 0.662–0.794) in both Youpwe and Manoka Island but no significantly difference was observed. Concerning the use of other strategies to prevent *Anopheles* bites, the level of anti-gSG6-P1 peptide IgG Ab response was significantly lower in individuals using strategies such as insecticidal twist or spray cans (ELISA median ΔOD: 0.693, 95%CI: 0.655–0.781) than those who did not in Manoka island (ELISA median ΔOD: 0.889, 95%CI: 0.835–1.031) with a significantly difference (p = 0.0021). In Youpwe, the level of IgG Ab response to gSG6-P1 peptide was not significantly different irrespective of additional measures taken to prevent *Anopheles* bites.

## Discussion

This study aimed to use a biomarker based on human IgG Ab response to *Anopheles* gSG6-P1 salivary peptide to assess in a preliminary study and for the first time, the level of human exposure to *Anopheles* bites in malaria endemic sittings in Cameroon, a Central African region. Specific IgG Ab response to gSG6-P1 peptide was detected in almost all individuals (99%) contrary to non-exposed foreigners (European negative control). This data suggest the use of this specific antibody response to gSG6-P1 peptide as a pertinent marker to assess human exposure to *Anopheles* mosquitoes in Cameroon. This finding is similar to those reported in other regions in Africa [23–29, 33, 34, 36–40], Americas [30] and Asia [31, 32],where it was shown that the IgG response to the saliva peptide was associated to the level of exposure to mosquito bites [25, 27, 28, 30, 37] and to malaria infectious status [23, 32, 40]. Together, our data and from these previous studies confirm the relevance of this candidate biomarker to be used to assess human exposure to malaria vectors worldwide. The specific IgG anti-gSG6-P1 response exhibited inter-individual heterogeneity, indicating that the exposure to *Anopheles* bites differ among individuals. Considering that, the similar data previously reported that this biomarker can discriminate the level of exposure to *Anopheles* between two individuals living in the same endemic area [23, 27, 29, 30, 32, 36, 40]. Our study reveals no difference in specific IgG response to the gSG6-P1 peptide according to the gender. Indeed, previously reported studies confirms that the Ab response to gSG6-P1 peptide could not detect human exposure to *Anopheles* bites regardless of the gender [24, 26, 30, 32, 37, 41].

Interestingly, the population of Manoka Island had higher levels of specific IgG anti-gSG6-P1 response than Youpwe. Several studies reported that the level of IgG Ab response to gSG6-P1 peptide is significantly associated with the level of exposure to *Anopheles* bites [23, 24, 26, 30, 36] and, previous study in these same two areas reported a significant higher aggressiveness of Anopheles mosquitoes in Manoka than in Youpwe [35]. This higher antibody response level may be due to the fact that rural characters of the Island of Manoka with natural water collections are more favorable to the proliferation of *Anopheles* as shown in our other studies conducted in these areas and where we also reported higher densities of *Anopheles* mosquitoes breeding site in Manoka Island than in Youpwe [35, 42, 43]. In these studies, we also found that the type of habitation in Manoka Island offer more possibility to mosquitoes to bite inside the house than in Youpwé [35, 42]. Together, these studies suggest that the high level of urbanization in Youpwe offer less favorable condition of Anopheles mosquitos’ development. Similarly, a high level of IgG response to gSG6-P1 salivary peptide was reported in rural and less urbanized areas compared to urbanized area in Senegal [40].

The level of the IgG Ab response to gSG6-P1 salivary peptide was previously reported to be positively associated with malaria infection status, *Plasmodium* infected individuals developed higher immune response than non-infected [23, 29, 30, 32, 34, 36, 40, 41]. Similar was made in the present study with the level of IgG Ab response significantly higher in *Plasmodium* infected individuals than non-infected. This reinforces the idea that, in addition to being a biomarker of human exposure to malaria vectors bites, the gSG6-P1 peptide could also be used as a tool for assessing the risk of malaria transmission and therefore a tool for predicting malaria morbidity as it was also previously observed in Senegal [40]. Furthermore, such indicator will be more important for an efficient estimation of malaria transmission intensity, especially in areas with a low level of transmission or in context of elimination of malaria where the densities of Plasmodium-infected mosquito are became very low.

Regarding the level of antibody response and malaria infection according to studies areas, there is significant difference between infected vs non-infected individual in Youpwe whereas there is no difference in Manoka Island (Fig 3B & 3C respectively). This discrepancy could be explain by the difference intensity of exposure level between studied sites (lower in Youpwe compared to Manoka Island according to previous results shown in Fig 2), suggested that peoples are less exposed to malaria vectors bites in Youpwe, where urbanization has considerable reduced mosquito breeding site and therefore the population of anopheles with consequent lower exposition to mosquito bites in Youpwe. Indeed, it has been shown in several previous studies with this saliva biomarker that it was an indicator of the transmission in area presenting a low exposure/ transmission [23, 40]. In contrast, no association between specific IgG level and infectious status (difference in IgG level between infected individual or no) was observed in areas with high exposition [28], this could indicate that most peoples are exposed to mosquito bites, most they will be infected, and as they are exposed to vectors bites at the same frequency, it will not be a difference within infected and non-infected individual as we observe in the Island of Manoka where people are most exposed to mosquitos bites regardless their living environment.

In contrast, to previous study in Senegal and Angola [34, 37, 38], where the level of IgG-anti gSG6-P1 response was less important in people reported sleeping on LLINs than in those who did not, we observed no such association with high level of the IgG-anti gSG6-P1 response and individuals reported to sleep under LLINs. A low level of IgG response to gSG6-P1 salivary peptide associated to with LLINs use was also reported in a study conducted in Angola and comparing this IgG response level in the same area before and post-large scale distribution of LLINs [38]. Due to the information given on the LLINs use by participants without a thorough observation, the findings reported by the above studies may be not observed in our study. Moreover, habitants in these localities spend more time out of their houses at night due to their main activities. Finally, it could not be excluded to be bite mainly outdoor compared to indoor i.e when they are not under bed-net, as reported in our previous study in the both localities [35]. In this study, we reported that the overall biting density of *Anopheles (An*.*) coluzzii* was 13, 96 b/p/n indoor and 34.76 b/p/n outdoor in Manoka, and 4,75 b/p/n indoor and 24,83 b/p/n outdoor in Youpwè and, also that density of *An*. *coluzzii* was significantly higher outdoor than indoor in the both localities [35]. However, our observation of the level of IgG Ab response to gSG6-P1 peptide significantly lower among individuals using the other strategies as coils and spray bombs to prevent *Anopheles* bites compared to those that do not is consistent with other studies conducted in Senegal and Angola [34, 38] and, that suggest the using of coils and/or spray bombs may reduce human-vector contact and subsequently help to prevent malaria transmission.

## Conclusion

The present study shows for the first time that a specific response to specific *Anopheles* salivary peptide gSG6-P1 were recognized by the different blood samples in Cameroon, with the highest reactivity observed among people with infection and living in the area with high endemicity level. Although our two study sites are geographically closed, environmental and ecological differences may result in a difference of exposition to Anopheles bites and therefore to malaria transmission. Thus, further studies including entomological, ecological and environmental data could allow having a better idea of the situation. These observations could be very important in the decision making regardless of the vector control strategies implemented by the NMCP. Moreover, given the complexity of the vectors distribution in Central Africa with secondary vectors (*An*. *nili*, *An*. *moucheti*, *An*. *rufipes*, etc.), it required to conduct another studies in the localities hosting these vectors and, to assess the potential of this biomarker candidate to also measure exposure to malaria secondary vectors.

## Abbreviations

ACTs: Artemisinin-based combination therapies
b/p/n: Bite/person/night
ELISA: Enzyme-Linked Immunosorbent Assay
EIR: Entomological inoculation rate
gSG6-P1: gambiae salivary gland protein 6-peptide 1
HBR: Human biting rate
HLC: Human landing catches
IgG: Immunoglobulin G
LLINs: Long lasting insecticidal nets
LTC: Light trap catches
M: molar
MTI: Malaria transmission intensity
NMCP: National Malaria Control Program
μL: microliter
OD: Optical density
P.f: Plasmodium falciparum
RDT: Rapid diagnostic test
WHO: World health organization
